# Infected host responses across entomopathogenic nematode phylogeny

**DOI:** 10.21307/jofnem-2021-105

**Published:** 2021-12-14

**Authors:** Hilal Erdogan, Glen Stevens, Asa Stevens, David Shapiro-Ilan, Fatma Kaplan, Hans Alborn, Edwin Lewis

**Affiliations:** 1 University of Idaho, Department of Entomology, Plant Pathology and Nematology, Moscow, ID, 83844; 2 Faculty of Agriculture, Department of Biosystems Engineering, Bursa Uludağ University, Bursa, 16059, Turkey; 3 USDA-ARS, Southeastern Fruit and Tree Nut Research Laboratory, Byron, GA, 31008; 4 Pheronym, Inc., Davis, CA, 95618; 5 Center for Medical, Agricultural, and Veterinary Entomology, U.S. Department of Agriculture Agricultural Research Service, 1700 SW 23rd Drive, Gainesville, FL

**Keywords:** Dispersal, Entomopathogenic nematode, Host cadaver, Infectivity, Macerate

## Abstract

We used a phylogenetic framework to examine the relationship between entomopathogenic nematode (EPN) vertical dispersal and infectivity when EPNs are exposed to a mixture of compounds found in late-stage EPN-infected insect cadavers. EPNs from five phylogenetically close and distant species (*Heterorhabditis bacteriophora*, *H. georgiana*, *H. megidis*, *H. indica* and *Steinernema feltiae*) were exposed to cadaver macerate produced by their own species’ infection and by *H. bacteriophora* infected hosts. We found that only three of the five species (*H. bacteriophora*, *H. indica* and *S. feltiae*) responded to exposure to their own macerate by increasing rates of dispersal. When we exposed all five species to a *H. bacteriophora* infected host macerate, we found that only *H. bacteriophora* responded by increasing dispersal, and that the most distantly related species (*S. feltiae*) essentially halted dispersal. These findings suggest that (1) responses to cadaver macerate vary, and (2) there may be a relationship between inherent dispersal rates and sensitivity to macerate exposure, as the most rapidly dispersing species (*H. megidis*) showed no response to macerate exposure.

Organisms are exposed to an array of cues and signals in the environment, to which their responses have a range of consequences in terms of fitness. Pheromones, for example, are chemical signals that change the behavior of conspecifics in ways that include attracting mates, establishing territories, or modifying foraging behaviors. Researchers have addressed the causes and consequences of pheromone diversity among related species across a broad range of systems (e.g., Symonds and Elgar, 2008; Groot et al., 2016; Salmon et al., 2019). Despite their importance to fitness, pheromones are a small part of the diverse suite of sensory information an organism may detect and perhaps respond to at any given time. It is the balance between ‘general’ sensory information (e.g., food cues or temperature and moisture gradients) and specific signals (e.g., pheromones) that likely drives organism responses. In entomopathogenic nematodes (EPNs), which are obligate parasites of insects, these cues and signals are encountered in two vastly different environments: inside the infected host and outside the host in the soil.

Across a group of related species, responsiveness to cues and signals could be predicted to be conserved (e.g., host location cues for parasites) or divergent (e.g., sex pheromone signaling for closely related species (Symonds and Elgar, 2008), depending on potential fitness outcomes. In the case of nematodes, a group of related compounds called ascarosides appear to function as pheromone communication systems across a range of nematode species that includes plant-parasitic nematodes (*Meloidogyne* spp.), the model bacterial-feeding *C. elegans*, and entomopathogenic nematodes from the families Steinernematidae and Heterorhabditidae (Kaplan et al., 2012). These ascaroside pheromones regulate dauer formation, aggregation, and various behaviors such as dispersal in *C. elegans*; additionally, extracts containing ascaroside pheromones have been shown to influence dispersal and infectivity of infective-stage juvenile EPNs (Hartley et al., 2019; Oliveira-Hofman et al., 2019; Shapiro-Ilan et al., 2019).

EPNs are obligate insect endoparasites that rely on a symbiotic bacterial association (*Xenorhabdus* spp. for *Steinernema* spp., *Photorhabdus* spp. for *Heterorhabditis* spp.). Infective-stage juvenile nematodes (IJs) penetrate their insect host and release cells of their symbiotic bacteria. It is the proliferation of bacteria that in combination with nematode-released toxins kill the insect, and the nematodes feed on the bacteria that multiply within the insect host. The IJs develop, mate (in the case of most *Steinernema* spp., whereas first generation *Heterorhabditis* spp. IJs develop into hermaphrodites), and pass-through multiple generations in an insect host until conditions in that host decline. At that point, a new generation of IJs containing an inoculum of their species-specific symbiotic bacteria leave the host to search *en masse* for a new host.

At the late stages of infection, insect cadavers contain a range of cues and signals, some of which influence IJ behavior. Presumably, cues such as reduced resource availability, the buildup of waste products and crowding, trigger IJ formation and dispersal. Laboratory rearing conditions using White traps (White, 1927) to collect IJs remove them from the vicinity of their host cadaver when they emerge thereby reducing infectivity and movement. However, using nematodes freshly emerged from hosts or reestablishing contact with the host cadaver recovers infectivity and movement (Shapiro and Glazer, 1996; Shapiro and Lewis, 1999; Shapiro-Ilan et al., 2003).

This study uses a phylogenetic framework to assess how responsiveness to macerate from an EPN-infected insect, which contains both general host cues and species-specific pheromones, varies across four EPN species with increasing phylogenetic distance from a fifth “reference” species, *Heterorhabditis bacteriophora*. We used a 2016 phylogeny (Spiridonov and Subbotin, 2016) that describes three clades within the genus *Heterorhabditis* (Fig. 1). We chose at least one species from each *Heterorhabditis* clade, two from the clade containing *H. bacteriophora*, and a single *Steinernema* species (*S. feltiae*). We assessed the responsiveness of five species to their own macerate, and then tested the responses of all five species to *H. bacteriophora*-derived macerate in a common experiment. We hypothesized that responsiveness to host cues would be conserved, while species-specific effects of pheromones would be more variable, and predicted that (1) all species should respond to their own macerate by increasing dispersal and (2) the strength of the response to *H. bacteriophora*-derived macerate would decline with increasing phylogenetic distance.

**Figure 1:**
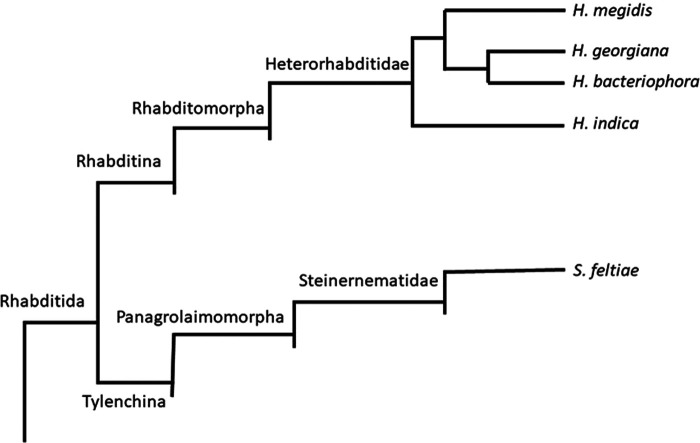
Abbreviated phylogeny showing the five EPN species used in the current study. Adapted from Kaplan et al. (2012), Spiridonov and Subbotin (2016) and De Brida et al. (2017).

## Materials and methods

### Rearing and culture of EPN

Cultures of five EPN species (*Heterorhabditis bacteriophora* Hb strain, *Heterorhabditis georgiana* Kesha strain, *H. indica* HOM1 strain, *H. megidis* UK211 strain, and *Steinernema feltiae* SN strain), were maintained in last instars of the greater wax moth, *Galleria mellonella* L. and collected using White traps (White, 1927). Infective-stage juveniles were stored at 14°C and tested within 7 to 14 d of emergence; additionally, all IJs used in the experiments were deconditioned within 10 d of emergence (Oliveira-Hofman et al., 2019) by triple-rinsing in DI water followed by 4d of storage at 14°C prior to addition to the columns.

### Dispersal assessments and macerate exposure

Dispersal was assessed using the protocol established in Wu et al. (2018). Stacked PVC columns were used to assess vertical dispersal of IJs. Columns were constructed of four stacked sections of PVC pipe (each section 4 cm inside diameter * 8.9 cm height). Columns were filled with sand at 10% moisture and a single last-instar *G. mellonella* larva (average mass ± std error 0.17 g ± 0.008 g) was added to the bottom of each column approximately two hours before IJs were added. Larvae were confined within an aluminum mesh screen envelope.

Immediately prior to addition to the columns, IJs were exposed to either host macerate or distilled water in the manner established in Wu et al. (2018). To prepare the macerate, cadavers were collected three days after the beginning of IJ emergence and homogenized with distilled water at the ratio of one cadaver: 1 mL DI water using a Tissue-Tearor (BioSpec Products, Inc., Bartelesville, OK, USA). Macerate mixtures were blended until thoroughly mixed. The mixture was centrifuged at 10,000 rpm for 45 sec (6,708 g) in an Eppendorf Minispin, and the resulting supernatant was removed to be used as host macerate. For an individual experiment, macerate samples were composited and frozen at -20° C for as long as 96 hr until the experiment was conducted; the day of the investigation, the composite macerate was thawed to room temperature, vortexed to mix, and sub-sampled for addition to dispersal columns. For macerate treatments, 5 mL of macerate was added to a suspension of 5,000 IJs in 200 µL of DI water (total 5.2 ml of macerate/IJ mixture); in treatments without macerate, IJs were suspended in 5.2 mL of DI water. IJ suspensions were incubated at room temperature for 20 min before adding to the top surface of the columns.

Columns were incubated at room temperature (21°  C) for 72 hr. After 72 h, columns were disassembled, and nematodes were extracted from the sand in each section by triple rinsing in distilled water. The number of IJs in each section was estimated by serial dilution. The number of IJs that had penetrated each *G. mellonella* larva was determined by dissection and subsequent counting using a stereomicroscope.

### Assessment of dispersal and host infection

We calculated a dispersal index using the midpoint of each column segment to estimate the average distance traveled per IJ over the 72-hr incubation period. IJs found within a segment were assumed to have traveled half the distance of that segment plus the total distance of any preceding segments; IJs that had penetrated the *G. mellonella* larva were counted as having travelled the entire length of the column. In addition to a dispersal index, we counted and assessed the effects of treatments on the number of IJs that penetrated (infected) each larva.

### Intra- and interspecific assays

We conducted these column assays under two sets of conditions (intraspecific and interspecific). In intraspecific trials, we assessed the dispersal of IJs after exposure to their own species macerate (that is, *G. mellonella* infected by conspecific IJs and homogenized). In interspecific trials, we assessed IJ dispersal of each of the five species after exposure to a common macerate derived from *G. mellonella* infected by *H. bacteriophora*. Intraspecific experiments (IJs exposed to their own macerate) were conducted 3 times, each time with a different culture batch of nematodes, with each time including 5 replicates of each species * treatment combination (150 columns total, 50 per iteration). Interspecific experiments (IJs exposed to a common *H. bacteriophora* macerate) were also conducted 3 times, each time with a different culture batch of nematodes; in this case, each time we established 2 replicates per species * treatment combination (60 columns total, 20 per iteration). Each of the eight iterations (five intraspecific, three interspecific) were conducted using different batches of IJs.

### Effect of exposure to *H. bacteriophora* macerate on *S. feltiae* mobility

Based on results showing a lack of dispersal when *S. feltiae* was exposed to *H. bacteriophora* macerate, we conducted a follow up examination in which approximately 5,000 *S. feltiae* IJs in 200 uL of DI water were exposed to either 5 mL of *H. bacteriophora* derived macerate or 5 mL of distilled water (the same rates used in the column assays). After incubation at room temperature for 20 min, IJs were added to a 100 mm diameter Petri plate containing 20 mL of 2% water agar. These nematode arenas were subsequently observed at post-exposure timepoints of 1, 2, 3, 4.5, 24, and 26 hr to determine whether exposure to *H. bacteriophora* macerate impaired mobility of *S. feltiae* IJs. At each observation timepoint, three groups of 25 IJs on each plate were randomly selected and assessed in terms of their movement: IJs were classified as moving (exhibiting sinusoidal movement), resting with some curve to their body (including a kinked tail), or completely straight. We used three replicate plates per treatment (macerate exposed vs. not exposed) and conducted two iterations of the six-plate experiment.

### Statistical analyses

Data were analyzed in R. We assessed responses of species to their own macerate and to the common macerate using two separate analyses of variance. As the effect of iteration was not statistically significant, data were combined across iterations for analysis. Due to significant departure from normality, rank transformation was used for analyses of variance; untransformed means are presented in all figures. Assessment of the effect of macerate exposure on invasion of the host was assessed using analyses of variance; Tukey’s HSD was used for post-hoc means separation assessment. The effect of macerate on *S. feltiae* mobility on agar was assessed using linear regression to model the effect of macerate exposure and time post exposure on the proportion of IJs that were moving. The effect of increasing phylogenetic distance from the reference species (*H. bacteriophora*) was judged by visual examination.

## Results

### Species’ responses to their own macerate

**Figure 2:**
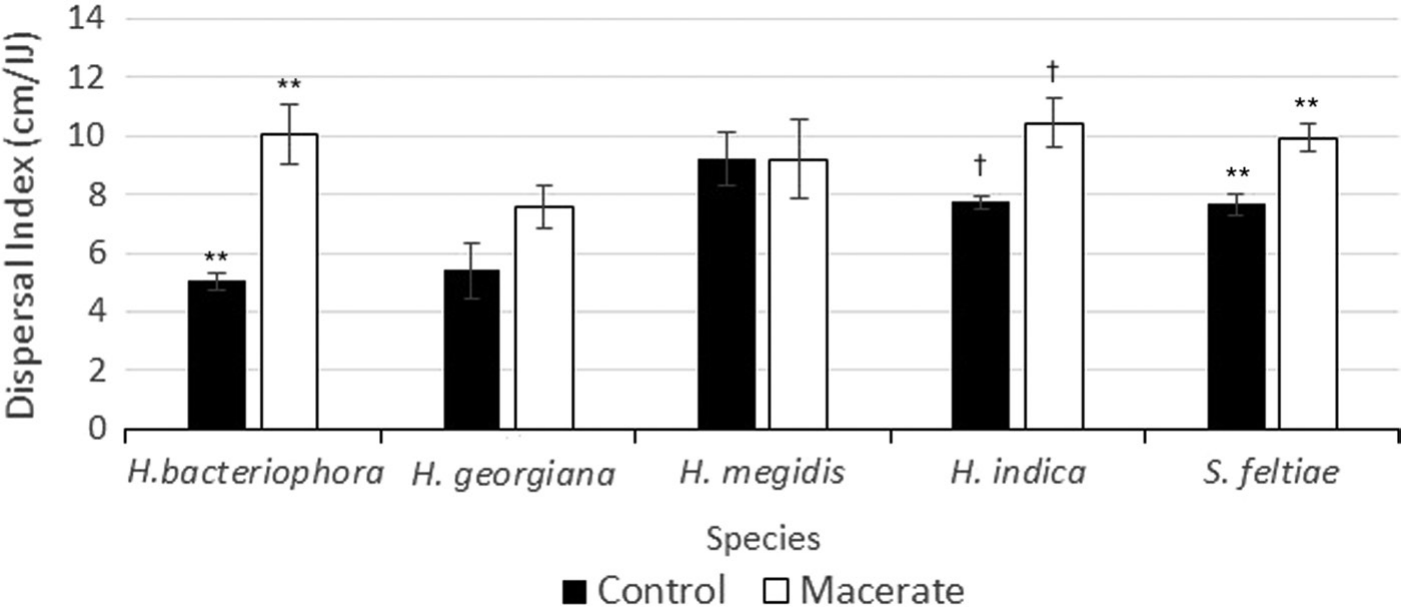
Dispersal responses of five EPN species after exposure to their own macerate. Bars show means +/− SEM. Symbols over bars indicate species that showed statistically significant increases in dispersal after conspecific macerate exposure († = *P* < 0.10, ** = *P* < 0.01).

Exposure of IJs to their own macerate increased dispersal downward through the column by about 40% on average across all species; this macerate effect was statistically significant (*F*_1,140_ = 13.6, *P* = 0.0003). The strength of this response varied significantly among the five species (*F*_4,140_ = 9.56, *P* < 0.0001). Dispersal of *H. bacteriophora* showed the strongest increase in response to macerate exposure (average 5 cm per IJ without macerate vs. 10 cm with macerate). Dispersal for *H. megidis* was the greatest on average of the five species regardless of macerate exposure (*P* < 0.0001); this dispersal appeared unchanged in response to macerate (Fig. 2), and the number of IJs invading the host was not significantly different for this species due to macerate exposure (*F*_1,28_ = 2.23, *P* = 0.15, Table 1). While dispersal of the other three species increased by between 30 and 40% after exposure to conspecific macerate; this difference was only significant (α < 0.05) in the case of *S. feltiae*.

**Table 1.** Average number of IJs infecting *G. mellonella* larvae after exposure to either DI water, their own macerate, or macerate derived from *H. bacteriophora* infected cadavers. Data show means ± SEM. Significant effects of macerate exposure based on post-hoc tests comparing DI water to macerate treatment; † = *P* < 0.10, * = *P* < 0.05.

Species	Own macerate^a^		*H. bacteriophora* macerate^b^	
	DI water	Macerate	DI water	Macerate
*H. bacteriophora*	0	4.4 ± 1.1*	0	3.2 ± 2.1
*H. georgiana*	0	15.9 ± 12.5	0.5 ± 0.5	0.8 ± 0.5
*H. megidis*	143.7 ± 31.1	83.7 ± 25.4	102.3 ± 50.2	65.3 ± 21.3
*H. indica*	2.4 ± 1.5	26.1 ± 13.4^†^	2.5 ± 2.5	1.8 ± 0.7
*S. feltiae*	0	8.6 ± 3.9*	0	0

Notes: ^a^*N* = 15 per cell. ^b^*N* = 6 per cell.

### Species’ responses to *H. bacteriophora* macerate

**Figure 3:**
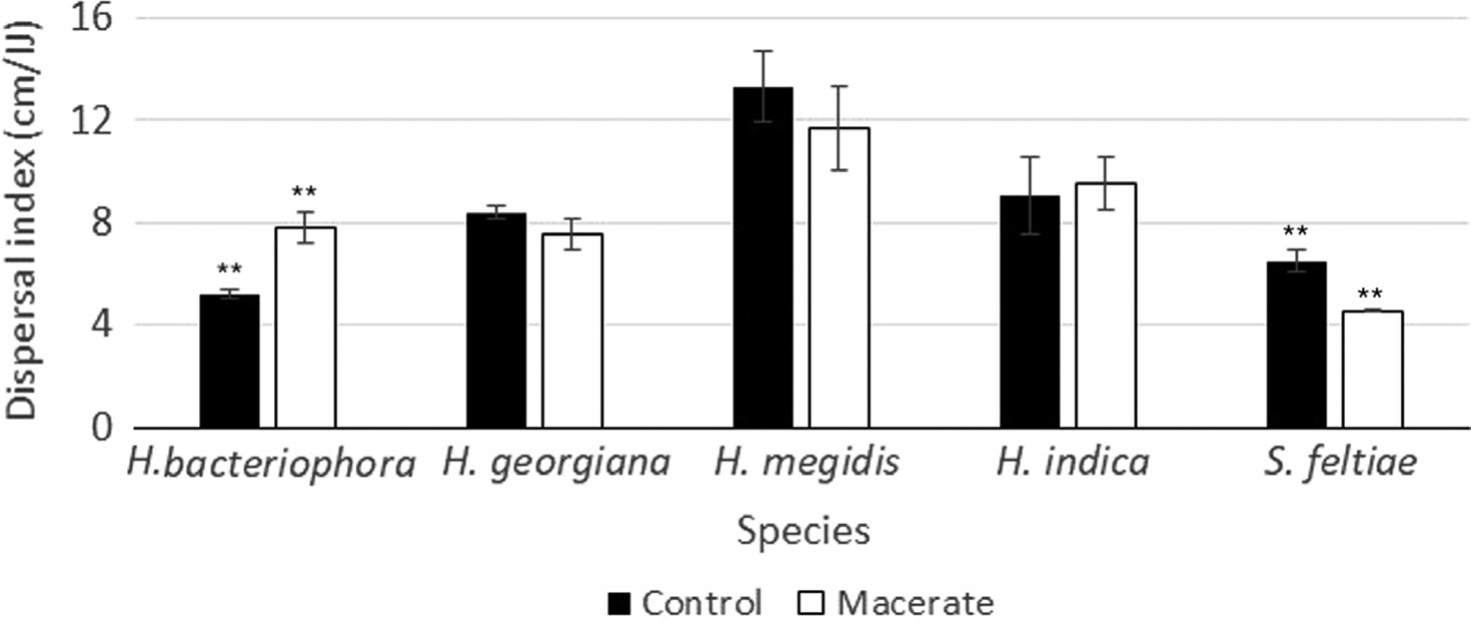
Dispersal responses of five EPN species after exposure to macerate derived from *Heterorhabditis bacteriophora*-infected cadavers. Bars show means ± SEM. Symbols over bars indicate species that showed statistically significant changes in dispersal after conspecific macerate exposure (**= *P* < 0.01). Relative to *H. bacteriophora*, increasing distance to the right along the graph represents increasing phylogenetic distance, according to Spiridonov and Subbotin (2016).

On average, while exposure of IJs to *H. bacteriophora* macerate significantly altered dispersal rates (*F*_1,50_ = 11.8, *P* = 0.001), the strength and direction of response to exposure varied among the different species (species effect *F*_4,50_ = 15.1, *P* < 0.001, species * macerate exposure interaction *F*_4,50_ = 5.87, *P* < 0.001). Dispersal of *H. bacteriophora* showed the only increase (average 5.2 cm per IJ without macerate vs. 7.8 cm with macerate), whereas the dispersal of *S. feltiae* declined by approximately 30% (Fig. 3). Dispersal of the three other *Heterorhabditis* spp. was statistically unchanged after exposure to *H. bacteriophora* macerate.

### Host infection responses to macerate exposure

Infection of *G. mellonella* larvae at the bottom of columns varied significantly across the different treatments. Within the conspecific assays (all species exposed to their own macerates) host infection rates varied significantly across the different species (*F*_4,139_ = 20.8, *P* < 0.0001); in addition, responses to macerate varied across the different species (species * macerate interaction *F*_4,139_ = 2.8, *P* = 0.026). Three of the species increased their rates of infection significantly when exposed to their own macerate (Table 1); *H. georgiana* did not show increased infection when exposed (*t* = -1.29, *P* = 0.22), and while fewer *H. megidis* infected the host in macerate than control treatments, this difference was not significant (*t* = 1.49, *P* = 0.15). Within the heterospecific assays (species exposed to *H. bacteriophora* macerate) there were differences among the species in the number of IJs that successfully infected (*F*_4,50_ = 12.2, *P* < 0.0001), but there were no significant effects of macerate exposure on infection (*F*_1,50_ = 0.99, *P* = 0.32).

### Response of *S. feltiae* to *H. bacteriophora* macerate

*S. feltiae* IJs were not quiescent (Kaplan et al., 2020) after exposure to macerate. Regression analysis of *S. feltiae* mobility over time post-macerate exposure showed that macerate-exposed IJs maintained a higher level of movement than control IJs over the 26 hr assessment (macerate exposure * time interaction was significant, *P* = 0.001, as was the overall regression model (*F*_3,56_ = 14.73, *P* < 0.0001)).

## Discussion

Our initial predictions were that that (1) all species would respond to their own macerate by increasing dispersal and that (2) the strength of the response to *H. bacteriophora*-derived macerate should decline with increasing phylogenetic distance.

The prediction that all species should respond to their own macerate was not supported. Post-hoc tests indicated that only three of the five species showed a significant increase in dispersal when exposed to macerate derived from conspecific-infected *G. mellonella* cadavers. Dispersal rates for *H. megidis* and *H. georgiana* did not differ between the distilled water and macerate exposure treatments, nor did they penetrate more *G. mellonella* at the bottom of the column. While *H. megidis* is known for rapid dispersal towards *G. mellonella* hosts (Boff and Smits, 2001; Boff et al., 2001), especially in sandy soil (Kruitbos et al., 2010), there are limited comparisons on the relative movement rates shown by *H. megidis* and other EPNs. *H. georgiana* showed baseline dispersal rates that were lower than *H. megidis,* but a difference in dispersal and infectivity in response to macerate exposure was not seen with either species with this assay.

Our results are largely in keeping with previous literature, though for the first time we find two species that failed to increase dispersal rates after exposure to their own (conspecific) host macerate. While Shapiro and Lewis (1999) did not observe an increase in infectivity by *S. carpocapsae* exposed to host macerate, other assessments showed enhanced dispersal of *S. carpocapsae* in response to macerate exposure (Shapiro and Glazer, 1996; Wu et al., 2018). Wu et al. (2018) showed that exposure to conspecific host macerate enhanced dispersal of *S. carpocapsae*, *S. feltiae*, and *Heterorhabditis bacteriophora*. In addition, Wu et al. (2018) observed that the response of *H. bacteriophora* to macerate exposure was stronger than that of *S. feltiae*, which is in keeping with what we observed.

Species’ strength of response to *H. bacteriophora* macerate did not decline with increasing phylogenetic distance. In general, while *H. bacteriophora* macerate stimulates *H. bacteriophora* dispersal, it had no effect on dispersal rates of congeners and essentially halted dispersal of an EPN from a different genus, *S. feltiae*. This may be a case of confusion between species-specific dispersal signals, which comprise part of the macerate, and the remaining host-associated cues such as the presence of bacteria or waste products of nematodes. As these nematodes are all members of the order Rhabditida, and they share specific ascarosides as pheromone components, crosstalk between heterospecific pheromone exposure- and infection-related cues might be expected, though the results are difficult to predict. The degree of the reduction in the case of *S. feltiae* was particularly surprising: few if any of the *S. feltiae* IJs that were exposed to *H. bacteriophora* macerate dispersed below the uppermost section of the column. The effect on *S. feltiae* was not due to induced quiescence (Hiltpold et al., 2015; Kaplan et al., 2020) nor the nematocidal products produced by Photorhabdus, as *S. feltiae* IJs exposed to *H. bacteriophora* macerate were alive when columns were disassembled after 72 hours and macerate-exposed IJs on agar maintained a higher level of movement than water-exposed IJs.

The lack of cross-reactivity to macerate differs from recent work that examined the response of the EPN *Steinernema carpocapsae* to methanol extracts of spent cadavers. These extracts have some common components, including the ascaroside ascr#9; Kaplan et al. (2020) observed that all extracts from 8 species of host cadavers stimulated dispersal of *S. carpocapsae* IJs as compared to water-exposed controls. There are key differences in approach between the two experiments: these were shorter-term agar plate assays (much like Hartley et al., 2019, below) and we used a crude macerate supernatant and did not follow this with a methanol extraction process. Methanol extraction certainly alters the relative abundance of pheromones vs. host signals by increasing ascaroside concentrations and may alter the relative composition of ascarosides. Furthermore, when *S. carpocapsae* IJ dispersal rate was quantified by Kaplan et al. (2020), the dispersal rate varied among pheromone extracts derived from different species of EPN. Oliveira-Hofman et al. (2019) showed that while crude macerate increased both dispersal and infectivity, the response to the pheromone extract alone was stronger; this suggests that cross-talk between cues and signals is produced by macerate exposure. Since pheromone extracts of each species only contain a subset of signals in the infected host, they provide a small window into how infected host cues are perceived. Additionally, we exposed five species to macerate derived from a single EPN species, rather than exposing one species to methanol extracts from seven. Regardless, these results call into question how consistently EPN species respond to cadaver derived extracts produced by other species.

The lack of cross-reactivity to macerate that we observed also contrasts with recent results reported by Hartley et al. (2019). Their study observed that cadaver extract (i.e., contents released from punctured cadavers) stimulated dispersal in each of the four EPN species tested, whether that extract was derived from a conspecific or heterospecific infection. They observed that *S. feltiae* dispersal was stimulated by exposure to *H. megidis* derived macerate, while we saw that *H. bacteriophora* macerate essentially halted *S. feltiae* dispersal. Key methodological differences may contribute to these contrasting results: the dispersal trials of Hartley et al. (2019) were short-term assays conducted on agar plates (rather than sand columns), a factor that can result in marked differences in experimental observations (El-Borai et al., 2011); additionally, IJs were stored for 2 days, rather than as many as 14 days in the current experiment, and the dose of the extract exposure was far lower (0.008 cadaver equivalents, rather than the 5 cadaver equivalents we used). Dose in particular is a difficult issue, as the dose of extract exposure that is most relevant to ecological conditions may or may not be the dose that is the most effective for altering IJ behavior in an applied setting. The choice of 5 cadaver equivalents in this experiment was the result of prior research that showed this rate to be effective at stimulating dispersal in *H. bacteriophora*, rather than a contention that a particular dose is the most relevant or ideal for EPN applications. Future research across a range of exposure concentrations, environmental conditions (temperature, soil type, etc.) and perhaps even host species may help understand these differences.

While ascaroside pheromones, which are produced throughout an EPN infection, have been shown to be a key element that can promote dispersal and infection (Kaplan et al., 2012; Shapiro-Ilan et al., 2019), those are far from the only compounds found in a late-stage infection. Nitrogenous waste products that are released from EPN infected hosts can alter attraction and repulsion (Shapiro et al., 2000); EPN appear to be attracted to low quantities (at levels similar to those found early in infection) and repelled by higher concentrations found later in the infection. The ammonia present in insect feces can inhibit EPN IJ responses to hosts (Grewal et al., 1993) and may trigger IJ emergence (San-Blas et al., 2008). Additionally, compounds such as prenol (3-methyl-2-buten-1-ol) appear to alter EPN behavior; recent assessments (Kin et al., 2019 and Baiocchi et al., 2020) observed that while all of the EPN species tested were repelled by prenol, only a subset of the species tested responded to prenol exposure by increasing rates of dispersal (Kin et al., 2019).

What drives species-specificity in signal responses is likely to be a complex combination of ascarosides, infection-derived cues and perhaps additional yet-to-be described signaling molecules produced by the EPN themselves. Pheromone-based communication channels are not always limited to conspecifics, as evidenced by cross-attraction of aggregation pheromones of bark beetles and eavesdropping on pheromone channels by predators (Hofstetter et al., 2012). The presence of cues produced by food or hosts, monoterpenes in the case of bark beetles, further complicates the drivers of behavior. Researchers have long known that the presence of general host associated cues, such as CO_2_, alters IJ dispersal and infection behaviors and that signals like ascarosides also cause changes in behavior.

Given the ability of EPN to exert strong top-down pressure in ecosystems (e.g., Denno et al., 2008; Ram et al., 2008) and their use as inundative biological control agents in agriculture (Georgis et al., 2006), understanding their behavior is key to understanding and harnessing their biology. Their mass production, which is commonly accomplished with *in-vitro* liquid culture, negates their contact with infection-derived materials which can impact their efficacy. Thus, these signals and cues associated with infected hosts could be an important additive to EPN products just prior to application to boost their performance. These signals might not have to be species specific since there is some evidence of cross-species activity of pheromone extracts (Hartley et al., 2019; Kaplan et al., 2020), but other still to be discovered compounds that impact EPN behavior might also affect the dispersal behavior.
